# Out of Sight, Out of Mind—Explaining and Challenging the Re‐Institutionalisation of People With Learning Disabilities and/or Autistic People

**DOI:** 10.1111/1467-9566.70009

**Published:** 2025-02-12

**Authors:** Jon Glasby, Justin Waring, Robin Miller, Anne‐Marie Glasby, Rebecca Ince

**Affiliations:** ^1^ Department of Social Work and Social Care University of Birmingham Birmingham UK; ^2^ School of Social Sciences and Humanities Loughborough University Loughborough UK; ^3^ Changing Our Lives UK

## Abstract

During the twentieth century, many countries underwent processes of ‘de‐institutionalisation’—closing ‘asylums’ for people with mental health problems, learning disabilities and dementia. Despite this, the UK has witnessed a subsequent process of ‘re‐institutionalisation’ with the creation of new public/private sector facilities providing ‘secure’ care to large numbers of people, who can be residents for many years with no sense of when they may leave. In 2023, 2035 people with learning disabilities and/or autistic people were receiving inpatient hospital care in England, with 54% in hospital for over two years. Drawing on the lived experience of people in hospital/families, and the practice knowledge of front‐line staff, this paper critically analyses why this process of re‐institutionalisation may be taking place. Our argument is that institutional forms of care have gradually been re‐introduced—despite the influence of neoliberal health policies that have previously aimed at deinstitutionalisation and self‐care—because some people are viewed as ‘too difficult’ to govern through the prevailing dispositive of self‐care, and therefore become the subjects of more disciplinary forms of power. Once in hospital, the primary routes to ‘escape’ require performative acts of ‘good conduct’ that give confidence to professionals of a person’s capacity for self‐government.

## 1 Introduction

In the late twentieth century, many countries underwent processes of ‘de‐institutionalisation’—closing ‘asylums’ or long‐stay hospitals for people with mental health problems, learning disabilities and dementia (Rogers and Pilgrim 2001; Jack 1998; Ramon 1996; Bartlett and Wright 1999). Despite this, the UK has witnessed a subsequent process of ‘re‐institutionalisation’ with the creation of new public and private sector facilities providing ‘secure’/‘locked’ care to large numbers of people, who can be residents for many years with no sense of when they may leave. In 2023, 2035 people with learning disabilities and/or autistic people were receiving inpatient hospital care in England, with 54% in hospital for over 2 years (NHS Digital 2023). Not only are such people deprived of their liberty, but there is evidence of abusive, inhumane and degrading treatment (Jagger and Harris 2024; see also Box 1). This is arguably one of the most fundamental, distressing and scandalous human rights issues of the modern care system, but notwithstanding public and political attention, it seems an almost forgotten sociological problem.

**BOX 1 Winterbourne View (BBC 2012).**

1 BBC One's Panorama showed patients… being slapped and restrained under chairs, having their hair pulled and being held down as medication was forced into their mouths.

The victims, who had severe learning disabilities, were visibly upset and were shown screaming and shaking.

One victim was showered while fully clothed and had mouthwash poured into her eyes.

Undercover recordings showed one senior care worker… asking a patient whether they wanted him to get a ‘cheese grater and grate your face off?’.

The abuse was so bad that one patient, who had tried to jump out of a second floor window, was then mocked by staff members.

Andrew McDonnell [an independent expert commenting on footage] labelled some of the examples seen on film as ‘torture’.

This paper critically analyses why this process of re‐institutionalisation may be taking place and how it is being experienced by people who reside in these hospitals, and those charged with their care. It draws on research that investigated barriers to leaving long‐stay hospitals for people with learning disabilities and/or autistic people (Glasby et al. 2024) and interprets the findings through Foucault's writing on disciplinary power and governmentality (Foucault 1991, 2008) to understand the role of health and care professionals operating at the nexus of these two modalities of power. Our argument is that locked/institutional forms of care have gradually been re‐introduced—in spite of neoliberal policies aimed at deinstitutionalisation and self‐care—because some people with learning disabilities and/or autistic people are viewed as ‘too difficult’ to govern through self‐care and therefore become the subjects of more disciplinary power. Once in hospital, the study shows how the primary routes to ‘escape’ institutionalisation require preformative acts of ‘good conduct’ that give confidence to health, social care and legal professionals of a person's capacity for self‐government. As such, the paper seeks to understand the social dynamics that explain how and why these people traverse these regimes of power, and in turn, make a significant contribution to research that examines the topologies of power in contemporary society.

### 1.1 De‐Institutionalisation and a ‘New Long‐Stay Population’

From the 1960s onwards, health policies in the UK have pursued a policy of de‐institutionalisation, the advent of which is often associated with the then Minister of Health's famous ‘water tower’ speech of 1961:

I have intimated to the hospital authorities… that in 15 years' time there may well be needed not more than half as many places in ‘hospitals for mental illness’ as there are today. Expressed in numerical terms this would represent a redundancy of no fewer than 75,000 hospital beds.

(Powell 1961)

The ensuing de‐institutionalisation agenda has been interpreted in the context of multiple social, technological and economic forces, including medical/pharmaceutical advances; changing societal attitudes; a series of horrific abuse scandals; and changing economic priorities and the desire to reduce public spending on institutionalised welfare (Rogers and Pilgrim 2001; Jack 1998; Ramon 1996; Bartlett and Wright 1999). The closing of the asylums therefore resonated with the ideological imperatives of both the radical left who were critical of institutionalised care and the economic right who were critical of an expansive welfare state. The 1980s saw a further acceleration in the de‐institutionalisation of care facilities with a growing emphasis on community‐based care and with the mixed economy playing a bigger role in meeting people's care needs, culminating in the passage of the 1990 NHS and Community Care Act.

For many former patients, leaving hospital could lead to a better quality of life and represent a more cost‐effective use of public resources (Knapp et al. 1992; Leff 1997; see also Welshman and Walmsley 2006). However, there were concerns about the extent to which patchy and under‐funded community services could support people with very complex needs, particularly in crisis—with some commentators concerned that a ‘new long‐stay population’ might emerge (Lelliott and Wing 1994; Trieman and Leff 1998). This group might have histories of criminal or violent behaviour, might end up being homeless, might experience multiple hospital admissions and/or might run the risk of finding themselves unable to be discharged back into the community.

Decades later, one such ‘population’ is people with learning disabilities and/or autistic people, many of whom might be seen as displaying behaviour that ‘challenges’ services, might also have mental health problems, may have experienced extreme prior trauma and/or may have been diverted into health services from the criminal justice system. In the early twenty‐first century, they have arguably experienced a barely noticed form of re‐institutionalisation, with a growing number of people admitted to or spend large amounts of time in a variety of institutional settings (see Glasby et al. 2024 for an overview of key trends and background policy).

Rather than the ‘asylums’ of the Victorian era, these beds might be in mental health hospitals, in various secure wards for people admitted from the criminal justice system, assessed as being a risk to themselves or others, or in so‐called ‘assessment and treatment units’ (referred to here as ‘long‐stay hospital’ settings as a shorthand). However, these beds—often ‘out of area’ (i.e., potentially a long way away from people's homes/family) and in the private sector (see, e.g., MacDonald 2018; Birrell 2018)—can quickly become filled with people whom the care system finds difficult to discharge and support in the community, with some being little more than ‘holding bays’ for people with nowhere else to go. While some might be in rural areas similar to the previous asylums, others are ‘hidden in plain sight’. As but one example, a particularly infamous unit (see Box 1) was on an industrial estate on the edge of a motorway, with commuters travelling past each day seemingly unaware of its existence.

Crucially, this process of re‐institutionalisation only really became apparent after an undercover reporter filmed widespread abuse at a unit known as ‘Winterbourne View’ in 2012 (see below for further discussion). As part of subsequent official reviews, it became apparent that some 3400 people were living in hospital settings—as if this form of service provision had sprung back up, almost without anyone noticing. Despite a period of shock and official pledges to end this situation, there were still some 2035 people with learning disabilities and/or autistic people residing in inpatient facilities at the end of October 2023 (more than 10 years after Winterbourne View) (NHS Digital 2023). The vast majority are detained under the Mental Health Act and/or may have been diverted from the criminal justice system (to be treated in hospital rather than punished in prison). Perhaps bizarrely, people with a learning disability and/or autistic people do not need to have a ‘mental health problem’ to be detained under the Mental Health Act but can effectively be locked up against their will if they are displaying “abnormally aggressive or seriously irresponsible conduct” (Mental Health Act 2007). As an indication of the interpretive flexibility at stake here, accompanying guidance states that:

Neither term is defined in the Act, and it is not possible to define exactly what kind of behaviour would fall into either category. Inevitably, it will depend on the nature of the behaviour and the circumstances in which it is exhibited, and also on the extent to which it gives rise to a serious risk to the health or safety of the person or others, or both.

(Department of Health 2015, 207)

### 1.2 Dehumanisation and the ‘Corruption of Care’

An extensive body of research shows that long‐stay care facilities can too easily be the sites of dehumanising and abuse treatment. In 1969, Morris' *Sociological study of institutions for the mentally retarded* revealed a bleak picture of *“barrack‐like”* life in dilapidated buildings, dormitories of over 60 people with beds only inches apart and only *“rudimentary”* sanitary arrangements, *“totally lacking in opportunities for privacy”* (p. 310). Although abuse can occur anywhere, J. Martin (1984) suggests that this risk is increased in isolated, closed institutional settings where there is poor leadership, staff shortages and inadequate training. In extreme situations, this can lead to a ‘corruption of care’ whereby maintaining order overshadows the value of dignity and care. While examples of this are rife throughout the various hospital scandals, such as Normansfield, Ely and others (J. Martin 1984), the same inadvertent process of dehumanisation lies behind everyday practices that were often considered ‘normal’ (Waring and Bishop 2019). To illustrate, a charity chief executive who trained as a *“mental subnormality nurse”* in the 1970s reflects on how no one seemed to be shocked by the conditions in which patients lived:

In those days patients were issued with a denim suit, they had no clothes or possessions of their own. There were nine inches between the beds in the dormitories. Everybody had a bath on a Saturday morning. Two of us would bath seventy people. There were two baths in one bathroom, with no privacy between them. Seventy men would line up down the corridor, naked, clutching their clean bundles of clothes and towel. That was presented to me as acceptable, and everybody did it.

(quoted in Brend 2008, 19–20)

While modern audiences might hope that such horrific treatment is a thing of the past, the continued abuse and dehumanisation of people with learning disabilities was shockingly revealed in 2012 when the TV documentary, *Panorama*, broadcast undercover footage of abuse at an ‘assessment and treatment unit’ known as Winterbourne View (Box 1). This was a private unit, commissioned by the NHS, to provide active, specialist, multi‐disciplinary care—but staffed and managed almost entirely by unqualified support workers, a number of whom were subsequently jailed for abuse (Flynn 2012).

Taking stock of the literature on long‐stay hospitals, our study aimed to produce new understanding about the reasons why people with learning disabilities and/or autistic people have experienced a period of re‐institutionalisation in the context of a broader shift towards de‐institutionalisation, how this institutionalised care might be experienced as dehumanising and what action might be needed for more people to leave hospital and lead more ordinary lives in the community.

### 1.3 Theoretical Framing

To explain, and ultimately challenge, this process of re‐institutionalisation, we draw on Michel Foucault's ideas of social power. With specific relevance to the topic of long‐stay hospitals, his early work focused on the social construction of ‘madness’, and his ideas have been influential to elements of the anti‐psychiatry movement (Crossley 1998; Bracken and Thomas 2010). Foucault's work examines how ‘regimes of truth’, or prevailing ways of knowing, define the subjects of which they speak and so position these subjects in relations of power with others and themselves. His historical studies of madness, illness and criminality argued that these ‘abnormal subjects’ were re‐defined in the modern age through the emergence of certain ‘disciplines’—scientific bodies of knowledge with associated technologies of disciplinary practice—that were themselves aligned with changing economic and political rationalities. In the modern era, these subjects are controlled through the asylum, the hospital and the prison.

Foucault's (1991) *Discipline and punish* advanced his understanding of disciplinary power and provided the departure point for his later works on ‘bio‐power’ (Foucault 2007, 2008). For Foucault, discipline is

A type of power, a modality for its exercise, comprising a whole set of instruments, techniques, procedures, levels of application, targets; it is a ‘physics’ or an ‘anatomy’ of power, a technology. And it may be taken over either by ‘specialized’ institutions… (house of correction), or by institutions that use it as an essential instrument for a particular end (schools, hospitals), or by pre‐existing authorities that find in it a means of reinforcing or reorganizing their internal mechanisms of power.

(Foucault 1991, 215)

Central to his understanding of disciplinary power is the capacity of experts, via their technologies of observation, comparison, categorisation and hierarchisation, to determine the boundaries between normal and abnormal behaviours, from which to regularise and discipline behaviours. His account of Bentham's panopticon remains an influential physical representation of this disciplinary power (Foucault 1991, 149). These ideas explain how social life is governed through the discursive classification and control of ab/normal behaviours and an associated array of social institutions, whether schools, workplaces, hospitals and long‐stay hospitals (Roberts 2005; Piro 2008; D. Martin et al. 2015). Although disciplinary power might be seen as functioning ‘over’ subjects, it is important to see it as flowing ‘through’ the embodied practices of discursively constituted subjects in which the ‘expert’ or ‘watcher’ is discursively constituted as much as the ‘inmate’ or ‘patient’, albeit with unequal positions of authority.

Foucault's later works suggested that social control is often more potent when internalised through behavioural norms and identities or subjectification. These ideas featured in his writings on ‘governmentality’ which characterised modern liberal governments as being concerned with the productive health of populations (Foucault 2008). Rather than governing social life through a sovereign state or disciplinary institutions, the liberal art of governing is realised through the constitution of subjects capable of governing their own conduct in line with prevailing political‐economic rationalities (Miller and Rose 2008). As such, governmentality is often associated with bodies of knowledge, technologies of calculation and pastoral practices that deal with the conduct of conduct (G. Martin and Waring 2018). For matters of health and health care, there has been a shift from disciplinary power to governmentality that is implicit in the de‐institutionalisation agenda witnessed in many developed countries and is further manifested in policies that emphasise self‐care and individual responsibility for health promotion (Petersen and Lupton 1996; Jones 2018; Miller and Rose 2008). In this context, people are encouraged to take responsibility for their own self‐care with the expectation that the home or community will take the place of the hospital or asylum (Dyck et al. 2005).

Foucault (2008) offers a conceptualisation of power in which the governing of subjects is realised through distinct, but importantly linked, modalities of sovereign, disciplinary and govern‐mental power. These three regimes function as an interlocking triangle and so a question that stems from this idea is how and in what circumstances do these different modalities of power become predominant, combine or transition. Following Collier (2009), this calls for a topological analysis that can account for interplay between the different forms of power, without reducing analysis to one form. To elaborate, we might ask about the circumstances in which self‐care fails to achieve productive health, thereby calling for more disciplinary power, or in what circumstances does disciplinary power become unnecessary because subjects are effective in governing their own conduct. In developing this analysis, attention is needed to the agency of social actors that sit at the nexus of discipline and subjectification (Waring and Latif 2017). These ‘nexus actors’ are involved in both direct forms of disciplinary intervention and behavioural sanction while also cultivating obedience and self‐government (Foucault 2008; Waring and Latif 2017). This also requires attention to the subjects of power and how they also act upon themselves or in opposition to behavioural norms.

The re‐emergence of long‐stay hospitals represents a relevant problematic of governance. On the one hand they represent a form of disciplinary power in which subjects are contained ‘out of sight’ from the general public, but at the same time, there is a parallel policy discourse, that resembles a form of governmentality, in which patients are expected to be empowered to attend to their own wellbeing in community settings with less reliance on costly hospital care. As such, people who reside in long‐stay hospitals are seemingly caught between two modalities of power, and through their relationship with different health and care professionals, the particular form of governing is realised. This paper examines how the mode of governing such people is determined in the practices of different health and care professionals.

## 2 Methods

This paper draws on the findings of a study from 2021 to 2022 that aimed to better understand why some people with learning disabilities and/or autistic people are unable to leave hospital and continue to experience potentially degrading care (Glasby et al. 2024). From this new insight, the study aimed to identify practical lessons so that more people can leave hospital and lead more ordinary lives in the community. While our initial focus was on lived experience and practice knowledge, our Foucauldian framing emerged during later stages of data analysis, when trying to make sense of these insights from a wider sociological perspective.

While the definitions of ‘learning disability’ and ‘autism’ are seldom set out in national policy documents, we adopted definitions provided by the English Department of Health (2001, 14) and the National Autistic Society (www.autism.org.uk/about/what‐is.apsx):

- ‘Learning disability’ includes the presence of: “*a significantly reduced ability to understand new or complex information, to learn new skills (impaired intelligence), with; a reduced ability to cope independently (impaired social functioning); which started before adulthood, with a lasting effect on development. This definition encompasses people with a broad range of disabilities.”*
- ‘Autism’ is “*a lifelong developmental disability which affects how people communicate and interact with the world.*”

### 2.1 Hospital Selection/Engagement

A qualitative case study approach was taken that involved longitudinal/sustained collaboration with three long‐stay hospitals. While choice of sites was to some extent opportunistic (willingness to grant access), sites from locations reflect differences in local affluence, ethnicity and rurality; a mix of NHS/independent sector providers; a mix of service models (forensic services, assessment and treatment units and different levels of security); and a range of people and experiences (a mix of male/female wards, people with learning disabilities and/or autistic people, people with experience of the criminal justice system and people with experience of seclusion/segregation).

### 2.2 Patient Participation

**FIGURE 1:**
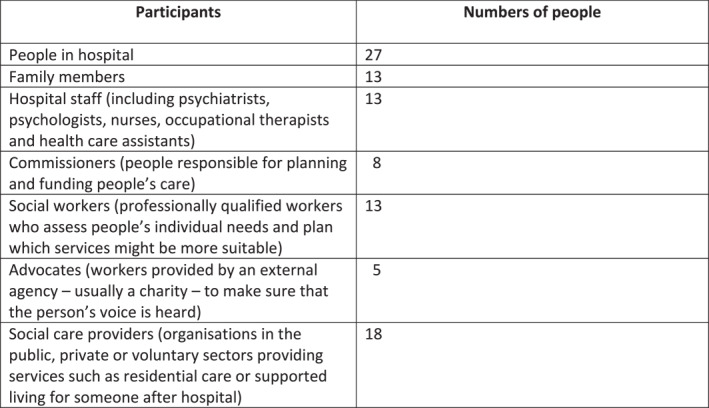
Participants.

Having secured NHS research ethics and local approvals, we worked with lead clinicians in each site to seek their professional opinion as to who could consent to take part and who might need a ‘consultee’ (usually a family member) under the Mental Capacity Act. These leads gave accessible introductory letters to everyone on the wards in question, with a reply slip to return to the research team if the person wanted to find out more about participation. Making sure to follow COVID guidance around hospital visitors, we based ourselves in 1–2 wards/units per site, interviewing everyone who agreed to take part. Subject to permission and depending on family circumstances, we also interviewed a family member and conducted an interview or focus group with a range of different care staff in different settings (Figure 1; see Glasby et al. 2024 for a detailed breakdown of people in hospital in terms of key demographics and length of stay, as well as for the number of different types of participants across different case study sites—given that this paper focuses on key themes from across our participants and sites, we have not provided this detailed breakdown here).

Our approach differed according to the ward and people we were working with. In some sites, we were located on the ward for several days, getting to know the service, minimising potential disruption and allowing people to approach us to find out more about the study. Where it was not possible to have such open access, a more formal approach was adopted (e.g., working with care staff, who would work with people on the ward to agree times to meet). People living on one ward also wanted us to attend their regular ‘patients’ council’ before deciding whether or not to participate. In all instances, we were extremely aware of the difficult circumstances people were experiencing and were flexible to ensure we spoke to people at a time that worked best for them, often returning to see someone on multiple occasions if they did not feel like speaking to us on a given day. We also spoke to ward staff, familiarising ourselves with people's preferred style of communication before beginning conversations.

During these interactions, we asked people about why they thought they were in hospital; what they wanted their life to be like/why they thought their life is not currently like that; and what would help them, or others, leave hospital more quickly. Where we had spoken to people early enough in the project, we repeated this 12 months later to explore what had changed (or not) over time. Where people did not communicate verbally, we used other forms of communication, such as ‘talking mats’ (www.talkingmats.com/) and art, utilising whatever communication mechanisms the person preferred (see Glasby et al. 2024 for details of creative approaches to communication). With permission from the participant, we also interviewed their family, observed multi‐disciplinary meetings and reviewed hospital case notes.

### 2.3 Staff Participation

We also carried out a focus group of hospital staff in each site, exploring how staff experience their work; how delays are brought about/what impact they have; and practical recommendations for change. These included front‐line members of the immediate ward/care team (e.g., support workers and nurses on the unit), as well as members of the wider clinical team (psychologists, psychiatrists, physios, OTs, social workers, etc). Focus groups enabled people from different professional backgrounds to interact and, wherever possible, reach a degree of consensus around key issues. To guard against staff feeling unable to speak freely, we also offered the opportunity to have an individual interview (while three participants chose this, it was more because they could not attend the focus group/still wanted to participate).

We also carried out online interviews with social workers allocated to each of our participants, as well as online interviews/focus groups with advocacy organisations and social care providers supporting people to leave hospital (recruiting both those active in our case study sites and from organisations on our advisory board/their memberships). Our interviews with social workers were also supplemented by national focus groups with social workers connected to the British Association of Social Workers' ‘homes not hospitals’ campaign.

### 2.4 Data Analysis

Interviews/focus groups were recorded using an encrypted recorder and professionally transcribed. We also kept detailed handwritten records of our time on the ward, insights from case notes and observations from review meetings, building up an in‐depth picture of each person and their situation. Where someone did not want the interview recorded or the discussion could not be transcribed due to the nature of the person's speech/communication, we drew on such notes (making sure that these were much more extensive than in situations where notes were only really accompanying the interview transcript).

Data were initially analysed using the framework approach (Ritchie and Spencer 1994), identifying key themes from the data and constantly checking back to refine emerging themes and ensure that these continued to represent the data (Miles and Huberman 1994). Initial codes were developed (by R.I., in discussion with J.G., R.M. and A.M.G.) after team meetings to share emerging informal themes (which were also sense‐checked with our advisory board and reference group). Final codes were agreed by consensus in regular team meetings, with two members of the team (R.I. and Frederick Konteh) each independently coding the same initial six interviews and meeting to compare their experiences and the outcomes of this process. In the latter stages of analysis, the identified themes were explored by all authors in relation to additional lines of theoretical interpretation with a number of sensitising perspectives identified, such as Goffman's work on total institutions and Foucault's disciplinary power. Through an iterative process, emerging themes were related to Foucault's ideas and conceptual tools as a means of developing a critical sociology of re‐institutionalisation.

In our findings section below, we indicate whether individual quotes came from a person with a learning disability (person with a learning disability 1, 2, etc.), a family member, a commissioner, an individual social worker or an advocate. Focus groups with hospital staff, care providers or social workers are labelled as ‘provider focus group 1’, ‘hospital focus group 2’ etc. While many quotes are from people with learning disabilities themselves, some come from family members (describing the situation of their family members). In many of these situations, the family members in question seemed to have a close relationship and were well placed to comment on the person's situation—we have not included quotes from family members speaking about a person where there were significant differences of opinion between the person and their family.

### 2.5 Reflections on Research Ethics/Conduct

We received research sponsorship from the University of Birmingham, ethical approval from a Health Research Authority Research Ethics Committee and local R&D approval from case study sites. The research team was experienced at conducting complex health and social care research in difficult environments, at working sensitively and ethically with issues of capacity/consent in ways that enable people who are seldom heard to take part in research and at working at the pace of individuals with particular communication needs. We conducted the research in a way that valued the voices and experiences of people with learning disabilities and/or autistic people, their families and front‐line staff while also recognising the need to minimise potential distress, be respectful of the complexities of life in long‐stay settings and ensure safety for everyone involved (e.g., in situations where people may have behaviour, i.e., labelled as ‘challenging’ services). To ensure that our research was informed by lived experience, we worked with a reference group of people with learning disabilities and/or autistic people and their families to co‐design our approach, sense‐check findings and support dissemination.

All participants were given information about the research (including in accessible formats) and we regularly checked that they understood study aims, consented to take part and were clear on key rights. Where a lead clinician assessed someone as unable to consent to take part, they approached a consultee on the person's behalf with an introductory letter, information sheet and consent form. Accessibility of materials was ensured by using images designed for people with learning disabilities from an accessible picture library, with input from a specialist charity working with people with learning disabilities. We also worked with our reference group to find the best way to explain the research when we were meeting people so that we answered people's questions as clearly and accessibly as possible.

## 3 Findings

### 3.1 ‘Difficult’ and ‘Ungovernable’ Subjects

People had complex journeys through services (Box 2), constantly moving between different parts of health, social care, educational and criminal justice systems. In general, services were set up to meet a series of specific needs, but the people in our study described themselves as not fitting neatly into the categories often used by the health and care sectors and the criminal justice system. For example, many families had repeatedly sought support when the person was young, feeling unable to cope with the distress that the person was experiencing but were routinely denied access to care until a crisis occurred and the person was admitted to a long‐stay hospital. One participant with a mild learning disability who had also committed a criminal offence described how it was a lottery as to whether they would end up in prison or hospital. While some people preferred being in hospital, others would prefer prison, where there is at least a sense of a scheduled ‘release date’. As this person suggests, there were tensions between health/social services and the criminal justice system as to how best to categorise and manage some people. While hospital staff would often better know people in terms of their complex needs, the Ministry of Justice was felt to operate at distance, only relying on indirect reports and making decisions which the person saw as arbitrary. Without understanding the circumstances of individuals, the criminal justice system was often viewed as resorting to custodial care typically on the basis of risk to self or community. In the quote below, the frustration of person 16, repeatedly turned down for the opportunity to try a period of unescorted leave from hospital, is clear:

They have written off for unescorted leave now… maybe six times and been turned down for every one… Because I’m sectioned, it has to go through Ministry of Justice so they’ve turned me down and hopefully I’ve just done a piece of psychology work which [they] asked for and so once that’s done, they’re going to write back off again and hopefully this time with that information, fingers crossed, definitely they might just say ‘yeah’. Because they’re just going to get bored with turning me down at one point.

(Person with a learning disability 16)

**BOX 2 Complex journeys.**

1

*I was in prison, then hospital, then back to prison, then hospital and then I stayed in hospital.*

(Person with a learning disability 2)

*The first time I was here was 9 years ago before I came back. And I was in there for six and a half years. And on here I was like three or 4 years. Then I've been on this ward, came back a second time for 2 years*.

(Person with a learning disability 26)

*I've been locked up about 14 years, 15 years. I've done about, maybe, about six in hospital… [It] started off when I was 18, and I'm just in and out of different prisons… I got diagnosed with schizophrenia and then [they] said that you need to come to hospital, so that's why I'm here now*.

(Person with a learning disability 19)

Another prominent issue was when people were diagnosed with a ‘personality disorder’ because this would lead most community services to refuse to work with the person, irrespective of their actual needs. The problems of categorisation not only impacted upon what types of facilities people were admitted to but how and when they could leave:

I’ve had about four places that have come to assess me but they’ve all turned me down… I don’t fit a criteria and because I don’t fit a criteria, the commissioners won’t accept me.

(Person with a learning disability 16)

It’s been the biggest thing that’s held him back since the diagnosis was changed… He has an existing diagnosis of ADHD he has from eight years old, whether he’s taking the medication or not to me is irrelevant, they didn’t seem to believe that he also had a diagnosis of ASD [autistic spectrum disorder] and asked if I could find the paperwork for that. I did say that you're his doctor, it should be on his NHS records… The biggest stumbling block that we've come across [is the diagnosis being changed] … He’s borderline LD because of the ADHD. The autistic spectrum disorder has just been left behind, it doesn’t get mentioned…. But now they’ve decided he’s borderline, so how you can be borderline ADHD I do not know… It’s affected [discharge] a lot because he’s not fitting anywhere. They did start looking at places for him to move on to go rehab… But the social worker he had couldn't do anything for him because… he didn’t meet the criteria… He got turned down for funding because he didn’t fit into any of these groups anymore.

(Family 16)

Somewhat paradoxically, when faced with such complexity and risk, many of the support packages discussed in multi‐disciplinary meetings resembled fairly standardised templates configured around pre‐defined categories. There was little evidence of personalised care, choice or control, such as via direct payments or personal budgets. Instead, most services seemed to work by assigning a label to the person in ways that fitted with the available service model or to avoid responsibility—even if it did not reflect the reality of people's lives. Once acquired, these labels were difficult to discard and often served to justify other agencies withdrawing or denying services:

Once people have, you know, got a certain history attached to them, then it becomes harder and harder to find providers. I think that is a major issue.

(Advocate 3)

I think what happens is people have a crisis and then instead of people recognising that’s a crisis and that’s a temporary thing, that becomes the person’s reality really, and it’s that reputation that just follows the person wherever they are.

(Provider focus group 1)

I think the biggest thing is there’s too many cracks in the services and it’s easy for people to say, ‘It’s not for my team’… I think people use diagnosis as a way of excluding people now.

(Commissioner for Person with a learning disability 16)

Such labels could become further cemented in long‐stay settings where feelings of anxiety and psychological distress were often made worse by stressful, noisy and chaotic environments. As the following person describes, their deprivation of liberty was a cause for emotional distress that when expressed reinforced ascribed labels of risk:

Imagine being trapped on a ward where you can’t just leave, I’d be frustrated… Just because you’ve got an index offence, then that makes you ‘risky’. If you shout they put a risk behaviour sometimes, or if they swear—we get frustrated as human beings, but because you’ve got an index offence and you’re in hospital, that makes it then more somehow.

(Hospital focus group 2)

[Some of the other people here are] thieves, scumbags and people who obviously ought to be in high secure… If you can prove you’re not a risk in this environment, you’ve done well.

(Person with a learning disability 5)

Beyond the issue of labelling, the challenges of categorisation stemmed from disputes about which agencies should be responsible if something went wrong. The following two quotes show how the insight and expertise of the responsible clinician were called into question by other actors:

The RC [Responsible Clinician], you know, who we’re led to believe is the all‐singing word of wisdom, can sometimes be extremely risk naïve.

(Provider focus group 3)

The provider accepted him, the local authority agreed the funding… and it was blocked by the RC… [The RC] just wanted him to go from medium to low secure then into the community… And I was trying to say to [the RC]… ‘give me the evidence that [B1] needs to be in low secure,… [B1’s] been in hospital eight years, what more do you want him to do’? And [the RC] was just saying ‘there are risks.’ I said ‘there will always be risks lifelong, it’s how we help [B1] manage to live with his profile’… It was like talking to a brick wall.

(Commissioner for Person with a learning disability 11)

The study therefore found a situation in which ‘the system’ found people with learning disabilities and/or autistic people difficult to categorise and more often saw them as complex and risky. This could prompt dispute about the most appropriate forms of support, but in light of concerns about risk, secure hospital care seemed to become the default model of care to mitigate risk and sidestep the differences in assessment manifested between health/social care and the criminal justice system. And as shown below, people often remained in these hospitals for much longer than anticipated because they continued to be seen as difficult to categorise in ways that enabled (self‐) care in the community.

### 3.2 Hidden in Plain Sight/Dehumanisation

Most participants had been in hospital for 1–2 years, five had stays of 4–5 years or more and two had been in hospital over 10 years. People came from all over the country with many placed in facilities a long way away from their communities and families, deepening a sense of isolation. Two of our case study sites were NHS organisations and one was in the independent sector, and while key themes were similar, one participant felt there were significant differences in the motivation of some public and private providers:

The NHS work to get you out—they don’t get paid to keep you there. It comes out of their budget, so they want to move you on. Private hospitals get paid to keep people.

(Person with a learning disability 5)

Although some felt being in hospital was helping them get well, many found it a very distressing experience with widespread boredom, frustration and fear. As the following participants describe, life inside had limited purpose, was disconnected from friends and family and offered little sense of meaning:

At the moment, because I've only been here for two months, it’s a bit slow… because it’s not just me here, so that staff, they have to do everything for different people, so at the moment it’s going slow, I'm not allowed out.

(Person with a learning disability 3)

We used to go onto the ward sometimes and there were residents and patients on the ward just on the corridor, just laid there, not even in their bedrooms and whatever, on sleeping bags… If he hadn’t have come out when he did he would have died because he were that bad.

(Family of Person with a learning disability 18, talking about a previous hospital)

To be honest with you, I just keep to myself, in my room, just keep away from everyone, because they’re always bickering and people kicking off.

(Person with a learning disability 19)

As one person explained, the environment of long‐stay care is often not conducive to prompting psychological wellness, and it can be easy for people to find themselves in a downward spiral and to miss the opportunities to find more appropriate care in the community:

You reach a peak of your health, so say like I'm well and I'm surrounded by unwell people, the behaviour of those unwell people would influence my well behaviour till I’m unwell, because the unwell people are more likely to be getting more attention and my progression ‐ like going out and stuff ‐ is being cut because people are kicking off and staff is needed… That’s just going to agitate you and then you're going to come from your peak to working downwards to being unwell again, then you're going to have to start from the beginning.

(Person with a learning disability 2)

Staff seemed just as aware of and frustrated by the very confusing and restricting nature of the system in which they worked. There was a sense that ‘normal’ rules and expectations do not apply and that staff who choose to work in these settings do so to make a difference despite all the difficulties:

Most people… have got the same core values. We’re very similar people. You wouldn’t be able to work here if you weren’t, it would be impossible.

(Social work focus group 1)

This value set we’re all talking about, you can’t teach it… I mean there’s stuff that we will put up with every day that would be absolutely intolerable to any community worker, because we have to, because we keep coming back to work.

(Psychologist, hospital focus group 1)

This environment is so specific, you know, there’s not many places you can go to work, have your ribs broken and then take the person [on a foreign trip] the next week. I’ve been in that situation so many times where people have broken my bones and yet I still take that positive risk with them because I know that deep down I can.

(Nurse, hospital focus group 1)

However, staff across all sites expressed extreme frustration at being unable to demonstrate such values, functioning more as a ‘holding’ service than a therapeutic one:

That sense of going round in circles or being constantly met with brick walls or dead ends when you’re trying your best for that service user to move them on… Sometimes it feels like, what we can actually do as nurses… is very little in the grand scheme of things. The mainstay of our… work is in that treatment phase, and then we find ourselves holding people and trying our best to keep them on an even keel while that wait is happening, and it can be quite difficult for the nursing staff to feel like they’re actually achieving anything.

(Nurse manager, hospital focus group 2)

The expectation… is that it’s a dynamic work environment ‐ short term, outcome‐focused. And the phrase that’s often used is, we’re just providing ‘bed and board’ now, you know? It’s just a B&B service, we’re hoteling people. We’ve trained to be nurses and occupational therapists, psychologists, whatever it is, but this person’s ready to go, so actually, all we’re doing is looking after them, and that’s different.

(OT, hospital focus group 2)

Overall, there was a sense of people being stuck in hospitals. For residents it often meant being far from family and home, despite often being close on the fringes of other communities. This sense of isolation and inertia was reflected in the experiences of staff who struggled with the limits on their ability to care and what seemed like an erosion of their core professional identities.

### 3.3 Finding ‘Escape Routes’

Following people up 1 year later was highly revealing, if depressing. Of the 21 people we were able to re‐contact, 15 were on the same ward, four had been discharged from hospital and two had been transferred to different wards in the same hospital. Of the latter, one person had moved from low to medium secure and one person from an assessment unit to a low secure ward. Thus, despite some people leaving hospital, most were exactly where they had been a year before, and two people were arguably even further away from leaving.

Although professionals were desperate to help people leave hospital, the barriers were formidable. On many occasions, staff (no doubt under significant pressure) made a series of blanket referrals to multiple community‐based service providers—essentially hoping that if they ‘scatter‐gunned’ (our phrase) for long enough, one of the referrals would eventually ‘stick.’ One social worker said she had applied to 15–16 places for someone she was supporting, and all said no. When asked why, the responsible clinician informed the team that this was due to historical ‘challenging’ behaviour issues and fears of incompatibility with their current residents.

A similar situation occurred with a person who had committed offences against children and was referred to a home which turned out to be near a school and inevitably withdrew the placement. One health‐care professional hearing our findings recounted a situation where someone had been rejected by over 80 different providers. As shown above, categorisation as ‘challenging’, historic labels and concerns of risk and responsibility made the idea of community‐based care difficult to realise.

The residents of long‐stay hospitals commonly believed that the way you ‘persuade’ people that you are well enough to leave hospital is through compliance with expected behaviours: if a professional suggests you do something, then you do it—otherwise you might be seen as ‘not cooperating’ or ‘lacking insight’. In difficult situations, sometimes chaotic environments, several people talked about *“keeping your head down”*, *“staying out of trouble”* and *“keeping yourself to yourself”* as a way of ‘avoiding trouble’ and maximising your chances of being discharged:

I suggest to people… to keep their heads down and to get on with the treatment. As soon as they keep their heads down and do the treatment, the quicker for them to get out of here.

(Person with a learning disability 20)

Obviously I don’t like it [here] but sometimes it’s alright if you kind of start realising that you stick to yourself and just behave yourself basically.

(Person with a learning disability 1)

[What keeps people in is] their behaviour. Saying stuff like, stupid stuff, keeping them back. Getting into trouble. Not sticking to the rules.

(Person with a learning disability 26)

This sense of needing to comply spilt over into the language people used, highlighting moral and normative behavioural expectations. For example, one person recently discharged suggested that this was because *“I was good.”* Another person was offered speech therapy and felt that they had to accept in order to be seen as ‘behaving’:

They want me to have it and [I’m] not allowed to… refuse stuff. I am allowed to refuse stuff but it looks bad on me because I'm refusing stuff… I don't think I've got—I can't say my ‘Rs’ very good… but a lot of it is because I'm from […]…, so I've got a different accent… But if it helps me get out it would be helpful ‐ but I'm not sure if it’s going to be helpful or not, because… it’s not because my speech is what I'm in here for.

(Person with a learning disability 3)

A prominent means of demonstrating behavioural compliance and the ability to look after oneself was when on ‘leave’ (whether in the hospital grounds or the community and whether escorted or unescorted). This was described as a process of incremental trust‐building but was far from straightforward. This was particularly the case if the person needed authorisation from the Ministry of Justice, which could be a lengthy and frustrating process. For example, one person described how they were ready to leave hospital for a place in a locked rehabilitation service, but the Ministry of Justice intervened and said they first needed more unescorted leave in the community. This leave was denied on three separate occasions (on the grounds that there was a risk of assaulting other people and of substance misuse), leaving the person stuck in a ‘Catch 22’ situation.

## 4 Discussion and Conclusion

Our analysis focuses on three themes that we suggest provide a foundation for a critical sociology of the re‐institutionalisation of people with learning disabilities and/or autistic people. Our first theme centres on how such people are diagnosed, categorised and labelled within the prevailing care system. Despite the overriding drive within policy towards (self‐) care within the community (NHS England 2019), the people involved in our study often presented with needs regarded as too complex to meet within an evolving community‐based model of care. These not only eluded diagnosis within the health and social care system but also the criminal justice system and arguably the education and housing systems. Although each system might be able to offer some partial categorisation, none appears sufficient to assume responsibility or to provide a holistic response. The sociology of diagnosis offers important insights for how clinical categorisation and labelling can represent a biographical disruption (Bury 1982) and the process of diagnosis can hasten the disappearance of the person (Blaxter 2009). Questions of risk feature significantly in diagnostic processes (Salter et al. 2011) and are inherent to the allocation of responsibility (Lupton 1999). Research in arguably less complex care settings shows how health‐care professionals can refuse jurisdictions where a case appears too complex or costly (Oh 2014) or where the risks of responsibility are too great (Waring and Bishop 2019). To some extent this echoes Beck's (1992) ‘organised irresponsibility’ or the idea that with some risk‐based decision‐making processes, it is difficult to identify an actor with overarching responsibility. In situations where a precise categorisation cannot be made and in the context of concerns with risk and allocation of responsibility, decision‐making processes appear to result in hospitalisation on the grounds that it can afford more detailed assessment and/or management of the associated risks. Relating these ideas back to the work of Foucault (2008), it might also be argued that the discursive expectations of constituting self‐care remain problematic for the people involved in our study and that it also remained difficult to assign a clear categorisation, and hence they become almost ‘ungovernable’. It is within this context that more extreme forms of disciplinary power—in the forms of re‐institutionalisation—are deployed until a categorisation can be found for transfer to another facility or into the community.

Our second theme shows how during their stay in hospital people can experience emotional turmoil, isolation and a loss of connection, reminiscent of Goffman's (1961) account of *Asylums*. The cutting of ties with the community and the erosion of personhood might explain accounts of abuse and inhumane treatment described in both the older literature and more recent public scandals (J. Martin 1984). Such processes are similarly reported in Waring and Bishop's (2019) account of hospital discharge which can all too often result in inhuman and unsafe care. Drawing on the Foucauldian‐inspired philosopher, Agamben (1998, 2005), they suggest some patients with complex needs are reduced to their ‘bare life’, thereby rendering inhumane noncare acceptable. Departing from Agamben, they suggest this arises not from sovereign authority over life or death but inadvertently through the fragilities of governmentality as expressed in the challenges of categorisation and the distribution of responsibility. In the context of long‐stay hospitals, similar processes might be at work. Looking at the experiences of staff, we found a parallel sense of despair and turmoil where ambitions to care are thwarted by the rules and procedures of the disciplinary system. In effect, caring professionals are reconstituted by the system as guards or wardens who are concerned with managing risk rather than supporting recovery. From this perspective, the corruption of professional duty from notions of care to noncare can be somewhat explained by the corruption of the professional identity by a system seemingly design to contain and conceal, with echoes of the Stanford Prison Experiences (Haney, Banks, and Zimbardo 1973; Zimbardo et al. 1973).

Our third theme considers how people can find a way out of long‐stay hospital. To some extent, this involves addressing the problems of categorisation outlined above, but this resembles a preformative act of compliance with categorical and behaviour expectations. Returning to Foucault's later works, one interpretation of this is that service users are required to create and display a self that is seen as less risky and capable of self‐care, thereby justifying the restoration of liberty and the reduction of disciplinary power. In effect, people need to become entrepreneurial in governing their own conduct as a gateway to being approved for self‐care in the community and discharge from disciplinary power (Foucault 2008). At the same time, professionals within these care systems need to find categories and care trajectories that might align with a person's unique circumstances and to which service users might themselves align. As shown above, this can be laborious work, navigating risks, making referrals and scheduling care, and thereby creating opportunities for such entrepreneurial behaviour. As such, it might be argued that the nexus of discipline and governmentality is itself ordered through the interlocking and recursive actions of multiple nexus actors.

Drawing together the above analysis, we might ask: why do some people with learning disabilities and/or autistic people find themselves residing and almost trapped in long‐stay hospitals ‘out of sight, out of mind’? The answer, we suggest, might be found in the difficulties society continues to have with understanding the complex circumstances of these people, where the existing apparatus of care is poorly configured to address their needs and where people are viewed as ‘unable to care for themselves’. As such, these people are hospitalised, seeming to acquire a type of archaic categorisation reserved for the ‘mad’ or ‘bad’ and finding themselves the subject of disciplinary control. In this sense, the care system operates at the nexus of disciplinary power and governmentality: trying to support people to be cared for or care for themselves in the community but, where questions of risk become too opaque, resorting to forms of disciplinary control that in themselves risk dehumanising and marginalising such people.

Taking a step back, while some services arguably let people down as children and as adults, failing to meet needs that do not fit into neat categories and incarcerating people almost indefinitely in difficult and distressing places, we were struck by the extent to which people's aspirations were often very simple and touching. At the end of the day, people valued their family and friends, liked animals, and just wanted to study, get a job and do things they enjoy:

I want this to be the last hospital or care home or prison ‐ so I’m trying to get it right this time round… Hopefully in a few months I’ll be out of here for good and I can just live life.

(Person with a learning disability 2)

Living on me own, having a job, settling down.

(Person with a learning disability 24)

Me and my friend last Saturday, we went out together. I was one‐on‐one, she was one‐on‐one and we went to Primark together, two charity shops, McDonalds and Poundland and this week or next week we’re meant to be going to the zoo together… I like the girls now, I’ll miss them but we’ve all got to move on, you know what I mean?… They’re all nice girls. We struggle but we have to be there for one another.

(Person with a learning disability 10)

This last quote is really revealing in its simplicity. While seemingly small in their own right, these acts of humanity made a major difference to people in a setting where they felt that so many of their rights and autonomy had been removed. In articulating a critical sociology of re‐institutionalisation, perhaps an antidote to coercion, labels and inhumane treatment is a very simple but powerful restatement of common humanity.

## Author Contributions

**Jon Glasby:** conceptualisation, funding acquisition, methodology, data curation, formal analysis, supervision, writing–original draft, writing–review and editing. **Justin Waring:** conceptualisation, data curation, formal analysis, writing–original draft. **Robin Miller:** conceptualisation, funding acquisition, methodology, data curation, investigation, formal analysis, writing–review and editing. **Anne‐Marie Glasby:** conceptualisation, funding acquisition, methodology, data curation, investigation, formal analysis, writing–review and editing. **Rebecca Ince:** data curation, formal analysis, investigation, writing–review and editing.

## Ethics Statement

This research is sponsored by the University of Birmingham and has been granted Health Research Authority (HRA) and Health and Care Research Wales (HCRW) Approval via Wales Research Ethics Committee 5 (IRAS project ID: 290750; protocol number: RG_20‐144; REC reference: 21/WA/0059)—approval received 19 February 2021.

## Consent

All participants in this study provided informed consent to take part in the original research and for anonymised quotes and examples to be used in subsequent publications.

## Conflicts of Interest

Jon Glasby declares being chair of the NIHR DLAF panel; recipient of a number of grants from NIHR and the ESRC/Health Foundation; NHS/local government nonexecutive director; member of the ARC WM advisory board and of various advisory boards for a series of publicly funded research projects; and senior fellow of NIHR School for Social Care Research/member of Birmingham SSCR leadership team. Robin Miller is the social care lead of West Midlands Applied Research Collaboration (ARC) (NIHR200165) and Co‐I on the National Social Care Priority Programme (NIHR200179); national ‘Demonstrator’ lead for IMPACT (ESRC/Health Foundation); Co‐I on other NIHR grants (e.g., NIHR135286—BRHUmB: Building A Research Hub For Palliative Care In Birmingham and the West Midlands); senior fellow of the NIHR School for Social Care Research; and chair of the Kent Research Partnership. Other authors have no competing interests.

## Further Information

Policy and practice resources from the study, including accessible versions, are available via: https://www.birmingham.ac.uk/schools/social‐policy/departments/social‐work‐social‐care/research/why‐are‐we‐stuck‐in‐hospital.

## Data Availability

This is a qualitative study about complex and very personal issues, and therefore the data generated are not suitable for sharing beyond those contained within the overall report. Further information can be obtained from the corresponding author.
